# Beyond the Driver Mutation: Immunotherapies in Gastrointestinal Stromal Tumors

**DOI:** 10.3389/fimmu.2021.715727

**Published:** 2021-08-20

**Authors:** Matthieu Roulleaux Dugage, Robin Lewis Jones, Jonathan Trent, Stéphane Champiat, Sarah Dumont

**Affiliations:** ^1^Département d’Oncologie Médicale, Gustave Roussy, Université Paris Saclay, Villejuif, France; ^2^Division of Clinical Studies, Institute of Cancer Research & Sarcoma Unit of the Royal Marsden NHS Foundation Trust, London, United Kingdom; ^3^Department of Medicine, Division of Oncology, Sylvester Comprehensive Cancer Center, University of Miami Miller School of Medicine, Miami, FL, United States; ^4^Département d’Innovation Thérapeutique et des Essais Précoces (DITEP), Gustave Roussy, Université Paris Saclay, Villejuif, France

**Keywords:** GIST - gastro intestinal stromal tumor, immunotherapy, PD-L1, imatinib, IDO - indoleamine 2,3-dioxygenase, KIT, immunologic response, macrophages (M1/M2)

## Abstract

Gastrointestinal stromal tumors (GISTs) are a subtype of soft tissue sarcoma (STS), and have become a concept of oncogenic addiction and targeted therapies.The large majority of these tumors develop after a mutation in *KIT* or platelet derived growth factor receptor α (*PDGFRα*), resulting in uncontrolled proliferation. GISTs are highly sensitive to imatinib. GISTs are immune infiltrated tumors with a predominance of tumor-associated macrophages (TAMs) and T-cells, including many CD8+ T-cells, whose numbers are prognostic. The genomic expression profile is that of an inhibited Th1 response and the presence of tertiary lymphoid structures and B cell signatures, which are known as predictive to response to ICI. However, the microtumoral environment has immunosuppressive attributes, with immunosuppressive M2 macrophages, overexpression of indoleamine 2,3-dioxygenase (IDO) or PD-L1, and loss of major histocompatibility complex type 1. In addition to inhibiting the *KIT* oncogene, imatinib appears to act by promoting cytotoxic T-cell activity, interacting with natural killer cells, and inhibiting the expression of PD-L1. Paradoxically, imatinib also appears to induce M2 polarization of macrophages. There have been few immunotherapy trials with anti-CTLA-4 or anti-PD-L1drugs and available clinical data are not very promising. Based on this comprehensive analysis of TME, we believe three immunotherapeutic strategies must be underlined in GIST. First, patients included in clinical trials must be better selected, based on the identified driver mutation (such as *PDGFRα* D842V mutation), the presence of tertiary lymphoid structures (TLS) or PD-L1 expression. Moreover, innovative immunotherapeutic agents also provide great interest in GIST, and there is a strong rationale for exploring IDO targeting after disease progression during imatinib therapy. Finally and most importantly, there is a strong rationale to combine of *c-kit* inhibition with immune checkpoint inhibitors.

## Introduction

Gastrointestinal stromal tumors (GISTs) represent a subtype of soft tissue sarcoma (STS) and are characterized by the malignant proliferation of Cajal cells in the bowel (1). Although rare with an annual rate of around 1 patients per 100.000 inhabitants, GISTs represent around 20% of STSs, making them the most frequent type of STS (2). Although they most frequently develop from the gastric stroma, GISTs can occur on every part of the digestive tract, and secondary locations are often liver and peritoneum (3). In most cases, the underlying mechanism is a mutation in the *KIT* gene (also known as *CD117*), coding for an activated transmembrane receptor c-kit and resulting in uncontrolled proliferation (4). Other cases are due to mutations in *platelet derived growth factor receptor α* (*PDGFR*α), *NF1*(coding for neurofibromin 1) or in the genes coding region for *succinate dehydrogenase* (SDH) (5). Treatment with imatinib results in deep (6) as well as sustained responses (7), but subsequent therapies offer a less durable clinical benefit. There is therefore an important need for new treatments for advanced GIST.

We conducted a literature review to describe the GIST microenvironment and current approaches to immunotherapy. The immune system seems to play a crucial role this controlling the disease, but the results of immunotherapy are disappointing to date. New molecular targets could be of interest.

## Gastrointestinal Stromal Tumors as a Specific Tumor Model

GISTs are a model of oncogenic addiction: its tumor cells are totally dependent on the activation of one molecular pathway, due to an identified mutation. Whereas some soft-tissue sarcomas are characterized by complex genomic variations (5) and are supposed to be more immunogenic, GIST oncogenesis is driven by a mutation in the *KIT* gene, coding for the transmembrane receptor c-kit (in 80% of all cases). This mutation occurs in exon 11 (coding for an intracellular domain), and more rarely, in exon 9 (coding for an extracellular domain). An activating *KIT* mutation leads to a signal for proliferation as well as the inhibition of apoptosis, through *phosphatidylinositol-3,4-bisphosphate kinase* (PIK3CA)/AKT/*mammalian target of rapamycin* (mTOR) and *mitogen activated proteins* (MAP) kinase pathways. *PDGFRα* is the second most frequent molecular alteration in GIST (in about 8% of cases), on various loci (such as D842V or V561D) and the D842V mutation is the most frequent alteration (8). The remaining 10-15% of tumors are *KIT/PDGFRα* wild-type, but several other mutations have been identified. SDH-deficient GISTs represent around 7% of all GISTs and are most frequent in young adults, occurring in around 50% of cases because of a loss-of-function germline mutation in one of the SDH complex genes (9). Mutations in the gene *NF1* can also be found, and autopsies of patients with Neurofibromatis 1 show undiagnosed GIST in one third of patients (10). *BRAF* V600E mutations have also been described in a small subset of patients, representing around 3.5% of all cases (11).

Advanced GIST is naturally chemoresistant with a response rate of about 7% to doxorubicin-based regimens (12). Prior to the introduction of imatinib and other tyrosine kinase inhibitors (TKIs), GISTs were associated with a very poor outcome with overall survival (OS) of only 12-19 months (13). However, the development of targeted therapy has revolutionized the prognosis of these patients. Imatinib is a multikinase inhibitor (multi-TKI) which was developed at the end of the 1990s and targets c-kit, PDGFRα, *Vascular endothelium growth factor* (VEGFR), *basic fibroblast growth factor* (b-FGF) among others kinases (14). Treatment with imatinib leads to progression-free survival (PFS) of around 30 months. The sensitivity of GISTs to imatinib mainly depends on the mutation locus and is higher in *KIT* exon 11 mutations (15). Unfortunately, not all GIST benefit from imatinib: SDH-deficient, NF1 and D842V-mutated GIST are imatinib resistant (5, 8, 9, 16). In imatinib-sensitive GIST, disease progression eventually occurs, mainly due to new oncogenic alterations. *KIT*-mutated GISTs can harbor secondary mutations in *KIT*, which most often occur in the imatinib target on c-kit, namely the *adenosine triphosphate* (ATP)-binding pocket (exon 13-14), or on the activation loop (exon 17-18) (5). In most cases, these mutations remain sensitive to sunitinib in a second-line setting or regorafenib in a third-line setting. Sunitinib is a multi-TKI targeting c-kit, PDGFRα and VEGFR, among others, which allows a meaningful median progression-free survival (median PFS) of around 6 months (17). After progression under sunitinib, regorafenib can be administrated, allowing a median PFS of around 5 months (18). Using all of these treatments sequentially results in a median OS of around 8 years in advanced GIST (15). More recently, ripretinib has been shown to result in median PFS of 6 months after three previous lines of treatments (19). This drug is currently being investigated as second-line *versus* sunitinib (20). The consensual strategy concerning advanced GIST is summarized in **Table 1**.

**Table 1:** Therapeutic options in the treatment of gastrointestinal stromal tumors (NCCN Guidelines, October 2020).

Phase	Setting	Treatment
Localized disease	(Neo-)Adjuvant	Imatinib
		*PGFRα-D842V:* Avapritinib
Advanced disease	First-line setting	Imatinib
		*PGFRα-D842V:* Avapritinib
	Second-line setting	Sunitinib
	Third-line setting	Regorafenib
	Fourth-line setting	Ripretinib
	Other options	Avapritinib
		Cabozantinb
		Dasatinib
		Nilotinib
		Pazopanib

Drug development in advanced GIST mainly focuses on new multi-TKI, with interesting activity (19, 21, 22), especially with the FDA (*Food and Drug Administration*) approval of avapritinib in D842V mutated GIST and ripretinib as fourth-line therapy. However, as in other tumor types, clinical benefit to systemic treatments decreases with the number of previous lines, and, in the very particular model of GIST, with the accumulation of resistance mutations. In the first-line setting, no TKI has improved outcome compared to imatinib. New treatment strategies are therefore needed.

Evidence is accumulating of an associated immune escape, leading to drug resistance and disease progression and this evidence opens up the field of immunotherapy for the treatment of advanced GIST.

## A Highly Infiltrated Tumor Microenvironment

Despite an oncogenesis based on a single pathway alteration and a low tumor mutational burden (23) suggesting a poor immunogenicity, GIST commonly harbors a rich immune infiltrate, suggesting a recognition of tumor cells by the immune system.

The microenvironment of GISTs is characterized by a high density of immune cells, with two main cell populations: tumor-associated macrophages (TAMs) and T-cells (CD4+, CD8+ and FoxP3+) in both untreated and treated tumors (24). There also seems to be some natural killer cells (NK cells) and a few B-cells. This microenvironment plays a major role in disease control, and *Rusakiewicz et al.* demonstrated that CD3+ cell and NKp46 cell infiltrates were independently positively correlated with PFS in both imatinib-treated and untreated localized GISTs, contrary to FoxP3 infiltrate (25). The type of *KIT* mutation did not seem to play a role in PFS in multivariate analysis. The worst prognosis was found amongst patients with a high Miettinen score but a low CD3+ cell count, and a low NKp46+ cell infiltrate.

The most common cells found in this immune infiltrate are TAMs, around twice more as T cells. M1 macrophages are differentiated from monocytes when exposed to *Granulocyte-macrophage colony-stimulating factor* (GM-CSF), *Lipopolysaccharide* (LPS) or *Interferon gamma* (IFN-γ) and promote an inflammatory microenvironment through the expression of IL-1, IL-6, IL-12 or *Tumor necrosis factor α* (TNFα). In contrast, M2 macrophages differentiate from monocytes in the presence of *Macrophage colony-stimulating factor* (M-CSF), IL-4 or IL-10 and are known to promote immune escape through the high expression of *Programmed death ligand* 1(PD-L1), IL-10 or *Transforming growth factor β* (TGFβ) (26). The polarization of TAMs is still controversial: in a cohort of 31 GIST samples with a majority of untreated primary tumors, these macrophages were in a majority of cases M2-polarized (27), whereas Cavnar et al. described an important M1 contingent in 25 untreated GISTs (28). In this study, TAMs became M2-polarized after treatment by imatinib (*see infra*). Although the most common T-cells are CD4+ helper lymphocytes, CD8+ T-cells are highly represented in this dense immune infiltrate. Regulatory T-cells (CD4+, FoxP3+) are also present but in much lower numbers (24). CD8+ T-cells are the key lymphocytes for killing tumor cells, and it has been proven that their presence is necessary to achieve a response to a treatment with anti-PD-1 (*programmed cell death 1*) antibodies (29). Furthermore, their density has been shown to be positively correlated with a response to immune checkpoint inhibitors (ICIs) in advanced melanomas (30) and renal cell carcinomas (31).

B-cells are described in GIST, but they seem to be present in higher numbers in metastatic lesions, where they represent around 2% of all immune cells, than in the primary tumor in untreated GISTs (32). The interest is rising regarding their importance in the immune response against cancer, where they play a role in tertiary lymphoid structures (*see* infra). Moreover, tumor infiltrating B-cells are known to provide a humoral antitumor response, leading to antibody-dependent cellular cytotoxicity (ADCC) and complement-dependent cytotoxicity (CDC) (25, 27).

As in other tumor models, there also seems to be immune activity mediated through NK cells. NK cells are lymphocytes belonging to the innate immune system and are involved in the first line defense against infection or tumors. They recognize pathological cells through a sum of activatory or inhibitory signals on their surface. They particularly target cells with a reduced expression of *major histocompatibility complex 1* (MHC I), which is common in GISTs. NK cells are described in the GIST microenvironment, and their presence is associated with a lower proliferation index and a better prognosis in untreated metastatic GIST (32). NK cells are activated by dendritic cells *via* the NKp30 receptor. However, in the peripheral blood of patients with advanced GIST, the NKp30c isotype is overexpressed at diagnosis. This isotype is the result of a splice variant due to genetic polymorphism and is immunosuppressive, in contrast to NKp30a and NKp30b. This leads to a decrease in TNFα, CD107a and IFNγ secretion, and seems to be associated with poorer OS (33).

Overall, with a tumor microenvironment highly infiltrated with different immune cells, whose proportion has a prognostic impact, the immune response seems to be of interest in GIST. Some studies have investigated the immune signatures in GIST more closely.

## An Inflammatory Profile Suggesting the Benefit of Immunotherapy

In a study analyzing the immune infiltrate of 31 patients with a majority of primary untreated tumors by RNA sequencing, Pantaleo et al. demonstrated that their tumor microenvironment is similar to that of melanomas, which is the very paradigm for efficacy of immunotherapy (27). The TIS (*T-cell inflamed signature)* encompasses 18 genes related to antigen presenting cell abundance, T-cell/NK cell abundance, IFN activity and T cell exhaustion and has been shown to be predictive for response to immunotherapy in melanomas (34) and head and neck carcinomas (35). TIS score for GIST was between the 65^th^ and 70^th^ percentile of the Cancer Genome Atlas dataset, which shows that there is an inhibited T cell activity as found in lung or renal carcinomas (27, 35). Interestingly, this signature was positively correlated with PD-L1 expression.

Based on the RNA-sequencing of 608 tumor samples of patients with STS, Petitprez et al. have recently investigated the role of tumor microenvironment (TME) in STS and its association with response to anti-PD1 immunotherapy. They created the Sarcoma Immune Classification (SIC), a classification that sorts STSs based on their tumor microenvironment, ranging from SIC-A (immune desert) to SIC-E (rich immune infiltrate).The main features of each group are described in **Table 2** (36). When applied to the pretherapeutic biopsies of 47 patients included in the SARC028 trial, SIC was found to be predictive of response to anti-PD-1 antibody therapy with around 50% of responders in the SIC E group. GIST is the most represented histologic subtype in this group, with around 25% of all 60 GISTs studied (*versus* around 20% in all sarcomas). This study highlights the role of B-cells in the immune response, with the importance of CXCL13 (an attractive TLS-associated B-cell chemokine) in the SIC-E group. As described above, B lymphocytes are part of the immune infiltrate in advanced GIST. Tertiary lymphoid structures (TLS) are ectopic lymphoid structures developing in non-lymphoid structures where intense and chronic inflammation takes place, including tumors. They are composed of a T-cell zone with mature dendritic cells and a B-cell follicle with a germinal center. More and more studies suggest their crucial involvement in antitumor immunity (37–41), where they seem to promote a T-cell response (42). Their clinical impact has also been shown in localized GIST, where they are very frequent (found in around 45% of patients) and seem to be positively correlated with a better OS and reduced risk of relapse (43).

**Table 2:** Tumor microenvironment features across the different groups in the Sarcoma Immune Classification (36).

SIC A	SIC B	SIC C	SIC D	SIC E
Immune desert	Heterogeneous low	Vascularized	Heterogeneous high	Immune and TLS high
**Low expression of immune cells-related genes**	Heterogeneously low expression of immune cells-related genes	**High expression of endothelial-related cells**	High expression of T-cell, B-cell, and NK-cell related genes	High expression of T-cell, B-cell, and NK-cell related genes
			High T Cell activation	High T Cell activation
			High MHC I expression	High MHC I expression B cell chemokine)
Low vasculature	Moderate ICP expression	Moderate ICP expression	High ICP expression	High ICP expression
Negligible CXCL13 expression	Low CXCL13 expression	Low CXCL13 expression	Moderate CXCL13 expression	**High CXCL13 expression and presence of TLS**

MHC I, Type I Major histocompatibility complex; TLS, Tertiary lymphoid structures; ICP, Immune checkpoint protein.

The impact of the driver mutation on tumor microenvironment (TME) remains controversial. In *Pantaleo et al*, no relationship was found between the identified mutation and TME (27). In constrast, *Vitiello et al.* found in a cohort of 75 untreated GISTs that *PDGFRα*-mutated GISTs were more infiltrated with immune cells, especially CD8+ cells, expressed more neoepitopes as well as regulatory T cell indicators and harbored a higher expression of ICP such as *T cell immunoreceptor with Ig and ITIM domains* (TIGIT), CD48 or *B- and T-lymphocyte attenuator* (BTLA) than *KIT*-mutated GISTs (44). This difference was even more important in *D842V*-mutated GISTs, which was corroborated by the comparison of RNA sequencing between 5 *D842V- PDGFRα* and 5 *non-D842V- PDGFRα* tumors (45). Immune control could explain the relatively low aggressiveness of these tumors.

These data provide a strong basis for the evaluation of immunotherapy approaches in GISTs.

## Mechanisms of Immune Escape in GIST

Some micro-GISTs (0.2-1cm) remain asymptomatic and will not evolve even if driven by the same oncogenic mutations as described above (46). This suggests the presence of other mechanisms of tumor development and progression to aggressive disease. Among other mechanisms, immune escape might play a major role.

### Immunosuppressive M2 Macrophages

As described previously, TAMs in GIST represent the most important immune cell subset in untreated GISTs and are often described as M2-polarized, thus promoting a rather immunosuppressive microenvironment (24, 27). Imatinib could accentuate this polarization (*see infra*) (2650).

### Indoleamine 2,3-Dioxygenase (IDO) Overexpression

**Figure 1:**
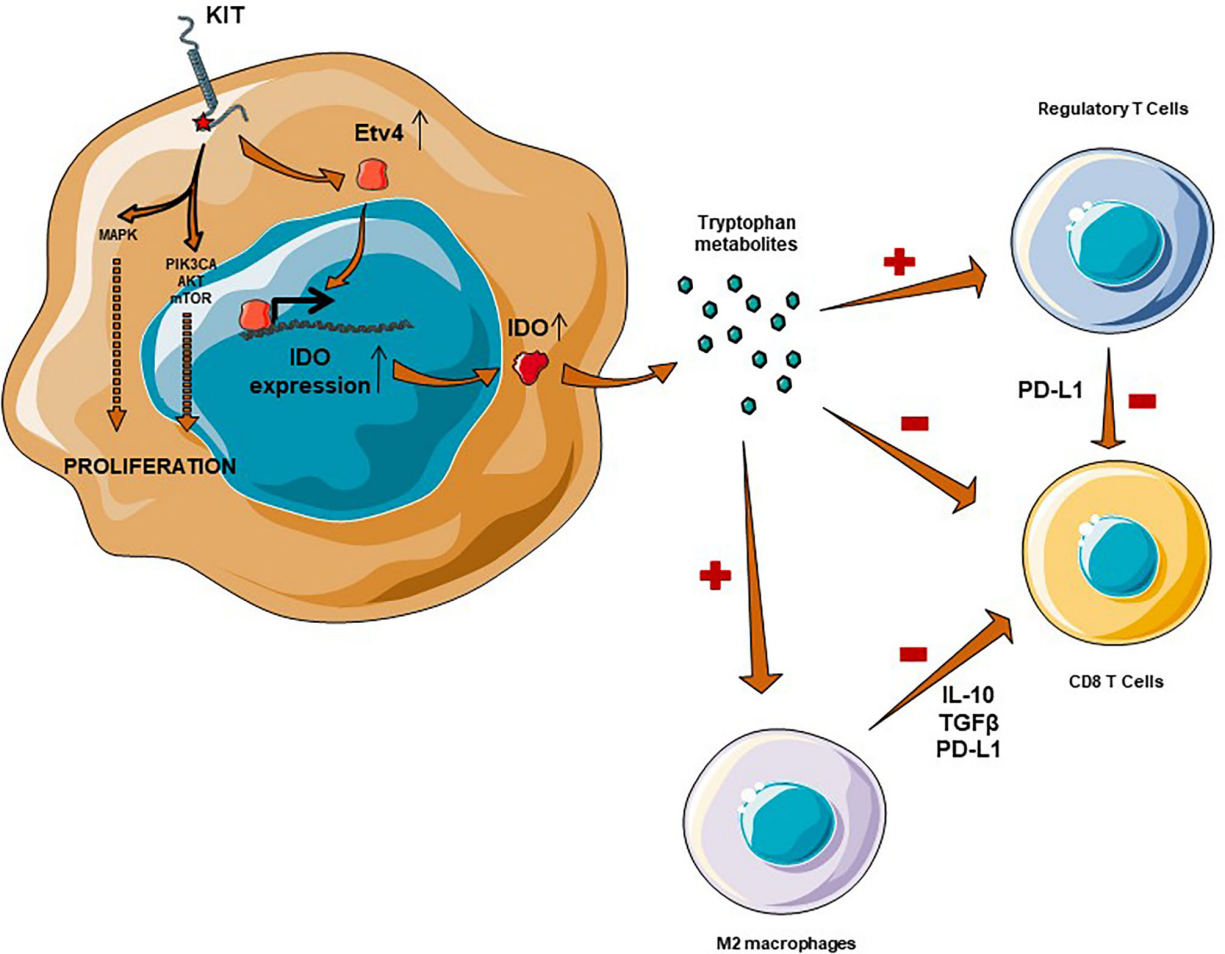
Immunosuppressive microenvironment in GIST is mediated by IDO and M2 macrophages. IDO expression is mediated through Etv4 and KIT activation, which results in an overexpression of IDO. IDO is responsible for a recruitment of regulatory T cells, an inhibition of CD8+ T Cells complementary to a macrophage M2-polarization.

The constitutional activation of c-kit induces, *via* transcription factor Etv4, the expression of IDO (47) (see **Figure 1**). IDO metabolizes the essential amino acid tryptophan into kynurenin, which is known to change the microenvironment from immunogenic to tolerogenic. IDO induces the differentiation of CD4+ lymphocytes into regulatory T lymphocytes and directly inhibits CD8+ T cells (48–50). Moreover, in the presence of tryptophan metabolites, antigen presenting cells (such as macrophages) are more likely to polarize to an immunotolerant phenotype, secreting TGFβ or IL-10 (51). In a Phase 2 trial evaluating the combination of pembrolizumab and cyclophosphamide in STS, IDO was overexpressed in 63% of cases in imatinib pretreated GISTs (52). The decrease in this ratio has been shown to be a major factor in the immune escape (24).

### Loss of MHC 1 Expression

Another crucial element of the immunosuppressive environment, described by Van Dongen et al, is the loss of expression of the MHC I, described in 70% of GISTs, leading to a decrease in the recognition of tumor cells by cytotoxic T lymphocytes. MHC I presents antigens on the surface of the cell, leading to antigen recognition by T-cells and antitumor immunity. This MHC I lower expression is well described in the immune escape of cancers and is often due to a loss of β2-microglobulin by tumor cells (53). Loss of MHC I is also an identified mechanism of secondary resistance to immunotherapy in melanomas (54).

### Immune Checkpoint Proteins Expression

Cytotoxic T-lymphocytes are inhibited by significant expression of immune checkpoint proteins (ICP). In comparison to circulating immune cells, tumor infiltrating lymphocytes (TILs) have a greater expression of PD1, *T-cell immunoglobulin and mucin containing protein-3* (TIM-3) or *Lymphocyte-Activation Gene 3* (LAG3) in imatinib-naive as well as imatinib-sensitive and resistant-tumors (55). This expression is independent of the type of mutation and seems to be increased in the case of resistance to imatinib. PD-L1 expression on tumor cells, described in about 70% of cases, has recently been identified as a poor prognostic factor in GIST and is inversely correlated with the presence of CD8+ T-lymphocytes (56, 57), suggesting a real lymphocyte anergy induced by PD-L1 expression on tumor cells. CD8+ T cells are also inhibited by regulatory T-cells and it has recently been shown that GISTs harbor a particularly high density of FoxP3+ T-cell-associated ICPs, such as *Glucocorticoid-Induced TNFR-Related protein* (GITR) or *Inducible T-cell costimulator* (ICOS). These ICPs are associated with a poorer outcome, underlining the role of regulatory T-cells in the immune escape of GIST (58).

## Immunological Effect of Imatinib

Imatinib is a TKI that targets c-kit and PDGFRα by interacting with the ATP binding site. However, in addition to an oncogenic addiction inhibition mechanism, accumulating evidence seems to point to immunologic activity.

On the one hand, it appears that imatinib, through the activation of CCAT enhancer binding protein β (C/EBPβ), is responsible for a reversible M2 polarization of macrophages (28). This effect is supported by the study by Van Dongen et al. which describes that M1 macrophages secrete IL-10 during imatinib treatment (24), and also by data showing that TAMs express less CD40 (59). Moreover, another off-target effect of imatinib is to inhibit differentiation and function of normal dendritic cells, as shown in a murine model (60).

On the other hand, the inhibition of c-kit by imatinib has a meaningful immunologic benefit in GISTs. First, imatinib seems to interact with NK cells as c-kit is located on the surface of dendritic cells and inhibits the cross-activation of NK lymphocytes. Imatinib, by inhibiting c-kit, induces NK cell activation and an increase in the Th1 response, with an increased secretion of IFNγ (61). This off-target activity seems to be relevant in terms of mechanism of action since the increase in secretion of IFNγ after 2 months of treatment with imatinib, which defines a group of “good immunological responders”, is a major prognostic factor (85% PFS at 2 years, *vs.* only 50% in non-responders) (62). It also appears that imatinib amplifies a pre-existing CD8+ immune response by inducing the influx of CD8+ T cells into the tumor and drainage node in a murine model, with decreased activity in the case of CD8 lymphodepletion (47). This influx is mainly related to the inhibition of tumor overexpression of IDO by imatinib, since a decrease in *IDO1* mRNA (independent of the decrease in the number of tumor cells) was mainly observed, leading to a depletion of intratumoral regulatory T cells and thus an increase in the CD8/Treg ratio. The decrease in this ratio has been shown to be a major factor in immune escape (24). This result is consistent with the analysis of human tumors, where imatinib-sensitive GISTs are enriched in CD8 T cells and have fewer regulatory T cells. The remaining question concerns resistance mutations and their implications for a recovery of IDO overexpression and eventually for imatinib escape. An additional mechanism suggested is the release of neoantigens by imatinib-induced lysis of tumor cells, with tumor cells in GISTs variably expressing peptides from the cancer testis antigens group (63). In addition, imatinib may decrease the immune escape by decreasing the expression of PD-L1 on tumor cells. The overexpression of PD-L1 induced by the presence of IFNγ is mediated by the *Janus kinase* (JAK)- *Signal Transducers and Activators of Transcription* (STAT) pathway and is blocked by the presence of imatinib (55). In models of chronic myeloid leukemia, imatinib has been shown to inhibit *vascular endothelial growth factor* (VEGF) transcription, through Sp1 and Sp3 transcription factors (64). VEGF is known to induce an immunosuppressive microenvironment, notably through a decrease in CD8/FoxP3+ T cell ratio and is a promising target in combination with immunotherapy (65). This inhibition probably has an important impact on the immunomodulatory microenvironment of GISTs by imatinib.

## Immunotherapy in GIST: Clinical Data

### Immune Checkpoint Inhibitors in GIST

Immune checkpoint inhibitors have been poorly explored in the management of GIST although, as discussed previously, preclinical data suggest they may be effective.

#### Anti-PD(L)1 Antibodies

Anti-PD(L)1 antibodies have not shown any efficacy against GIST as a monotherapy (**Table 3**). The Pembrosarc trial was a multicentric phase II trial evaluating pembrolizumab in combination with metronomic cyclophosphamide in advanced STS (52). The results were not encouraging in GIST: out of nine cases of GIST, there was no objective RECIST (*Response Evaluation Criteria in Solid Tumours*) response and 3 patients only had a stable disease as best overall response. PFS at 6 months was only 11%. In this study, the authors highlight the relevance of targeting IDO, as the tumor infiltrate was enriched in M2 macrophages overexpressing IDO in 63% of GISTs. In the preliminary results of a randomized phase II trial evaluating nivolumab or nivolumab and ipilimumab, 15 heavily pretreated patients with advanced GIST received nivolumab as a monotherapy: no partial responses were observed and the median PFS was 8.57 weeks (68). Seven patients had a stable disease as their best response, resulting in a clinical benefit rate of 46.7%. Alliance A091401 is a multicentric randomized phase II trial evaluating nivolumab alone or in combination with ipilimumab in advanced soft-tissue sarcomas. The results of the expansion cohorts were presented in 2020. In the 9 patients with GIST received nivolumab alone, the results were disappointing: no partial responses were observed as well, and the median PFS was 1.5 months.

**Table 3:** Results of clinical trials evaluating immunotherapeutic approaches in GIST.

Description	Phase	Number of GISTs	ORR (RECIST)	Median PFS	mOS	Notes	Reference
Peg-IFNα2b + imatinib followed by imatinib maintenance	II	8	100%	NR (> 3years)	NR	New PR achieved after reintroduction of peg-IFNα2 in a patient who progressed on imatinib maintenance therapy	Chen et al, 2012 (66)
Dasatinib + Ipilimumab in advanced GIST and other sarcomas	Ib	20	0%	2.8M	mOS: 13,5M	7/13 evaluable GISTs had PR by CHOI criteria	D’Angelo et al, 2017 (67)
Pembrolizumab + Cyclophosphamide in advanced STS	II	9	0%	6M-PFS: 11%	–	63% of GISTs showed a high IDO expression	Toulmonde et al, 2018 (52)
Nivolumab +/- ipilimumab in advanced GIST refractory to imatinib	II	N: 15	N: 0%	N: 8.57w	–	–	Singh AS et al, 2018 (68)
		N+ I: 12	N+I: 8.3%	N+I: 9.1w			
Nivolumab +/- Ipilimumab in advanced STS	II	N: 9	N : 0%	N : 1.5M	N: 9.1M	*-*	Chen et al, 2020 (69)
		N + I: 9	N+I : 0%	N+I : 2.9M	N+I :12.1M		

ORR, objective response rate; median PFS, median progression-free survival; mOS, median overall survival; STS, soft-tissue sarcoma; 6M-PFS, 6 month progression-free survival; N, nivolumab; N+I, nivolumab+ipilimumab; M, months; w, weeks.

#### Anti-CTLA-4 Antibodies

Anti-CTLA-4 (*cytotoxic T-lymphocyte-associated protein 4*) antibodies have not, to our knowledge, been studied as monotherapy in GIST.

In 2011, while describing the immunological effect of imatinib, Balachandran et al. suggested its synergy with anti-CTLA-4 antibodies (47). This synergy has not yet been observed in the clinic. In a phase Ib trial, the combination of dasatinib (multi-TKI with an anti-KIT activity) plus ipilimumab (anti-CTLA-4 antibody), 20 extensively pretreated patients with GIST were enrolled. This association did not demonstrate any efficacy (67): median PFS was 2.8 months and median OS was approximately 13 months. There appeared to be no response according to RECIST, but of the 13 evaluable cases, there were seven responses according to Choi criteria, which are known to have a better positive correlation to OS and PFS in GIST (70). Once again, one of the crucial elements of the immunosuppressive environment in GIST was IDO. Of 6 patients with evaluable biopsies, the only patient who had a loss of IDO expression following dasatinib and anti-CTLA-4 therapy had a stable disease for 19 weeks. Two patients without IDO suppression had progressive disease at first evaluation. One patient with SDH-deficient GIST had a stable disease for 47 weeks, without IDO suppression, but can reflect the natural history of this indolent subtype.

#### Association of Anti-PD-1 and Anti CTLA-4 Antibodies

The trials evaluating PD-1 and CTLA-4 antibodies coinhibition have also proven disappointing. In 2019, Singh AS et al. reported on 12 patients treated with nivolumab and ipilimumab after progression under imatinib in a phase II trial (68). One patient achieved a partial response, and 2 patients had a stable disease as best overall response. The median PFS was 9.1 weeks. Similarly, *Chen et al.* reported on the results of nivolumab in association with ipilimumab in the A091401 phase II trial (69). Nine patients received the combined therapy, and no objective response was observed. Median PFS was 2.9 months in this cohort, and median OS was 12.1 months. In comparison to the median overall survival of 9.1 months with nivolumab alone, the association seems to increase survival. However, the number of patients was not powered for overall survival, and the absence of objective response to both nivolumab and combination therapy did not support synergy. Once again the issue of the relevance of RECIST to evaluate PFS in GIST is apparent as is the importance of maintaining KIT inhibition when treating GIST with immunotherapy.

### Other Immunologic Approaches

An interesting approach has been to combine imatinib with pegylated IFNα2b(*peg-IFNα2b*). In a non-comparative monocentric phase II trial, eight patients with advanced imatinib-naive (or who had progressed more than 10 months after the end of adjuvant imatinib) GIST were treated by *peg-IFNα2b* weekly for 22 cycles in combination with imatinib, followed by imatinib maintenance. The safety profile was acceptable. The combination therapy resulted in an increase in IFNγ-producing lymphocytes, both in peripheral blood and inside the tumor. This immunological shift was responsible for an impressive 100% response rate, and lasting responses. Median PFS was not reached but no patients had disease progression before 2 years of treatment. Interestingly, after 3.6 years of median follow-up, the only patient who had tumor progression on imatinib maintenance monotherapy achieved a new partial response after the re-introduction of peg-IFNα2b (66).

## Perspectives and Promising Study Designs: The Need for Combination Therapies

With the combination of a rich inflammatory infiltrate, an inhibited Th1 response, identified mechanisms of immune escape and the demonstration of an immunologic effect of the main systemic therapy, exciting perspectives are opening up in the world of immune-oncology of GIST, a disease with an unfavorable evolution after the development of resistance to TKIs.

**Figure 2:**
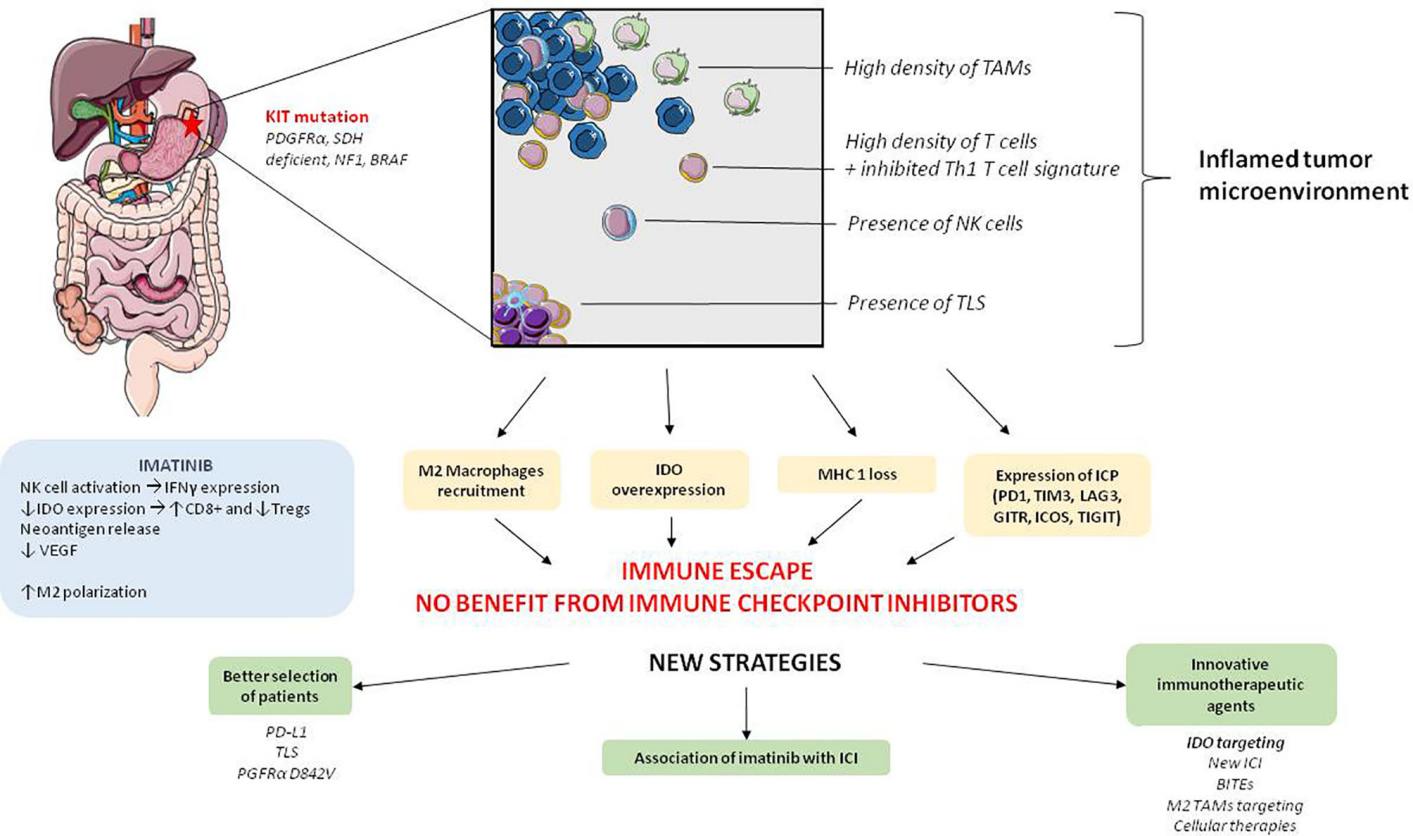
Global characteristics of tumor microenvironment and immunotherapeutic perspectives in GIST. PGFRα: platelet derived growth factor receptor α, SDH, succinate dehydrogenase; NF1, Neurofibrimin 1; NK cell, Natural killer cells; IDO, Indoleamine 2,3-dioxygenase; MHC, Major histocompatibility complex; ICP, Immune checkpoint protein; VEGF, vascular endothelium growth factor; IFN- γ, Interferon- γ; TLS, Tertiary lymphoid structure; PD-1, programmed cell death 1; TIM-3, T-cell immunoglobulin and mucin containing protein-3; LAG-3, Lymphocyte-Activation Gene 3; GITR, Glucocorticoid-Induced TNFR-Related protein; ICOS, Inducible T-cell costimulatory; TIGIT, T cell immunoreceptor with Ig and ITIM domains); BITEs, Bispécific T cell Engager antibodies; ICI, immune checkpoint inhibitors.

In spite of this, clinical trials evaluating anti-PD-(L)1 antibodies alone or in combination with anti-CTLA-4 antibodies have failed to demonstrate any efficacy in GIST so far. However, some responses or sustained stable diseases were described and recent translational studies in the field should encourage us to persevere: closer characterization of the immune infiltrate, frequency of TLSs, and immunologic classification of sarcomas (*see* **Figure 2**). In 2019, Zhao et al. demonstrated *in vitro* that imatinib was less effective in patients with high PD-L1expression, but there was a benefit of adding an anti-PD-L1 antibody in this population (56). Moreover, data are accumulating in favor of the early introduction of immunotherapy in the tumor course (71). Future trials evaluating anti-PD(L)1 should therefore focus on the first- or second-line setting and on the biological approaches, for example an evaluation of anti-PD(L)1 antibodies by selecting patients with a better chance of benefiting from these drugs: higher PD-L1 expression on tumor cells, patients with *PDGFRα* D842V mutation or classified in the *SIC-E* group (36).

Innovative immunotherapeutic approaches could also be of interest in GIST, and some are currently being investigated. One of them is to activate T cells in contact with tumor cells thanks to bispecific T-cell engager antibodies. A trial is currently evaluating XmAb18087, an antibody targeting CD3 and SSTR2, a surface antigen expressed by tumor cells in GIST (72). Moreover, even though the results of clinical trials evaluating IDO inhibitors have been disappointing to date in other tumors (73), targeting IDO in GISTs is of great interest considering its oncogenic overexpression. Epacadostat is currently being studied in combination with pembrolizumab in GIST (**Table 4**). We believe that the most promising strategy would be to study IDO inhibitors in combination with imatinib, following progression during imatinib monotherapy, in order to inhibit IDO-mediated immune escape. As discussed above, TAMs play a key role in the immunosuppressive TME and may be involved in tumor escape in GISTs. One strategy could be to promote their intratumor maturation and activation, and a CD-40 agonist antibody could allow better CD8+ T lymphocyte activation, while inhibiting imatinib-induced M2 polarization. In an *in vivo* model, the combination of imatinib with a CD-40 agonist provided better anti-tumor activity than imatinib alone, while there seemed to be effective activation of TAMs (59). In addition, a number of therapeutic approaches are currently being developed to target M2 macrophages such as STING (*Stimulator of Interferon Genes*) agonists (74), or anti-CLEVER-1 (*Common lymphatic endothelial and vascular endothelial receptor-1)* antibodies (75). Cellular therapies also seem interesting, but although Katz et al. succeeded in developing a 1st and a 2nd generation modified T-Cell with a KIT-ligand combined with an intracellular activation domain, no clinical study using such a strategy has been conducted so far (76).

**Table 4:** Ongoing studies of immunotherapy in gastrointestinal stromal tumors.

Study number	Phase	Description
**Association of anti-PD(L)-1 and anti-CTLA-4 antibodies**		
NCT02880020	Phase II	*Nivolumab +/-ipilimumab after progression under imatinib*
NCT02500797		
NCT02834013		
NCT02982486		
**Association of anti-PD-1 antibodies and IDO inhibitors**		
NCT03291054	Phase II	*Pembrolizumab +/- epacadostat after progression under imatinib*
**Association of TKI and anti-PD(L)1 antibody**		
NCT04258956	Phase II	*Axitinib + avelumab after progression under imatinib*
NCT01738139	Phase I	*Imatinib + ipilimumab in tumors with a c-kit mutation*
NCT03609424	Phase I/II	*Imatinib + PDR001*
**Bispecific T Cell Engagers antibodies (BITEs)**		
NCT03411915	Phase I	*XmAb18087 (bispecific antibody against anti-SSTR2 et CD3)*

Eventually, there are strong arguments pushing to evaluate anti-PD-(L)1 in combination with imatinib. Imatinib enhances IFN-γ secretion by NK cells, lowers VEGF and IDO expression in TME, thus resulting in an influx of CD8+ T cells and a decrease of regulatory T cells. Moreover, it seems unreasonable not to target KIT or PGFRα mutations in a disease in which oncogenic addiction plays such an important role. This supposition is corroborated by the work by Chen et al, and the impressive 100% response rate to imatinib combined with peg-IFNα2b (66). Based on these observations, it would be interesting to combine anti-PD(L)1 and imatinib treatment, before immunologic escape of the tumor, in a first-line setting. Therapeutic trials are currently exploring the relevance of inhibiting KIT and PD(L)1 pathways concomitantly (see **Table 4**), but to our knowledge, no association evaluates such an association with imatinib. With regard to the activity of anti-CTLA-4 antibodies, the synergy reported by *Balachandran et al.* has not been demonstrated clinically with the combination of dasatinib plus ipilimumab (67). However, dasatinib has been less studied for its immunological impact than imatinib. A phase 1 trial evaluating a combination of ipilimumab and imatinib is currently underway (see **Table 4**). Finally, as described above, some other ICP lead to T cell exhaustion and to immune escape in GIST, such as LAG-3 or TIM-3, and could be of interest in combination with imatinib. ICI targeting regulatory T cells, such as GITR agonists or ICOS, also seem promising in this setting.

Overall, this review summarizes the rationale to evaluate immunologic therapeutics in GIST, the paradigm for oncogenic driver mutation, and the limits of current investigative approaches. We believe three approaches must be highlighted: a better selection of patients included in clinical trials (presence of TLS, PD-L1 expression, *PDGFRα-*D842V mutation), the use of innovative immunotherapeutic drugs (especially IDO inhibitors), and most importantly the combination of *c-kit* inhibition with immune checkpoint inhibitors. One of the limits of this review is that we chose to focus on therapeutics which are developed specifically in GISTs and thus restricted the field of promising therapies. On the other hand, we think the comprehensive analysis of TME in GIST we provide and its correlation in terms of treatment strategies might help drug development in this very particular disease.

## Conclusion

The GIST microenvironment is highly infiltrated with immune cells, with a large infiltrate of CD8+ T-cells (associated with a genomic signature of inhibited Th1 immune response), the presence of B-cells and TLSs, and NK cell activity. Despite this inflammatory infiltrate, however, an immune escape is observed, mediated primarily by the recruitment of immunosuppressive M2 macrophages, secretion of IDO by tumor cells, recruitment of regulatory T cells, loss of MHC type 1 and expression of ICPs.

Imatinib has demonstrated immunologic activity in the management of GIST and appears to promote a CD8+ T-cell response. However, the results of clinical trials of immunotherapy treatments (anti-PD(L)1 and anti-CTLA-4 antibodies) after progression during imatinib treatment have been disappointing to date.

Promising perspectives are based on a better selection of patients (presence of TLS, PD-L1 expression, *PDGFRα-*D842V mutation), innovative therapeutic agents (especially IDO inhibitors) and the association on immunotherapeutic agents with imatinib.

## Author Contributions

MRD, RLJ, SC and SD contributed to conception of this review. MRD wrote the first draft of the manuscript and designed the figures and tables. All authors contributed to the article and approved the submitted version.

## Acknowledgments

We thank Newmed for providing a language review of this work.

## Conflict of Interest

The authors declare that the research was conducted in the absence of any commercial or financial relationships that could be construed as a potential conflict of interest.

## Publisher’s Note

All claims expressed in this article are solely those of the authors and do not necessarily represent those of their affiliated organizations, or those of the publisher, the editors and the reviewers. Any product that may be evaluated in this article, or claim that may be made by its manufacturer, is not guaranteed or endorsed by the publisher.
